# Interleukin-6 and C-reactive protein/albumin ratio as predictors of COVID-19 severity and mortality

**DOI:** 10.1186/s43168-021-00054-1

**Published:** 2021-01-14

**Authors:** Mohamed El-Shabrawy, Maha E. Alsadik, Maher El-Shafei, Ahmed A. Abdelmoaty, Ahmed S. Alazzouni, Marwa M. Esawy, Marwa A. Shabana

**Affiliations:** 1 Chest Medicine Department, Faculty of Human Medicine, Zagazig University, Zagazig, Egypt; 2 Medical Microbiology and Immunology Department, Faculty of Human Medicine, Zagazig University, Zagazig, Egypt; 3 Tropical Medicine Department, Faculty of Human Medicine, Zagazig University, Zagazig, Egypt; 4 Histology and Histochemistry, Faculty of Science, Helwan University, Helwan, Egypt; 5 Clinical Pathology Department, Faculty of Human Medicine, Zagazig University, Zagazig, Egypt

**Keywords:** COVID-19, C-reactive protein/albumin ratio, Interleukin-6, Mortality, Severity

## Abstract

**Background:**

Coronavirus disease 2019 (COVID-19) was announced in early December 2019. The pandemic situation is declared. This study aimed to evaluate the role of biomarkers in estimating the severity and predicting the prognosis of COVID-19.

**Results:**

A total of 116 confirmed patients were included in this study. The patients were evaluated clinically. The disease severity was assessed. The measured and calculated laboratory tests were done. The primary outcome is the 30-day mortality. Patients were assigned to the severe (14.7%) and non-severe (85.3%) groups. At IL-6 level of 32.3 pg/mL (the highest Youden’s index = 0.77), IL-6 can differentiate severe from non-severe patients with 82.4% sensitivity and 94.4% specificity. IL-6 can predict the severity [odds ratio of 87.7 (95% CI = 18.9-408.2) (*P* < 0.0001)]. After adjustment to the significant clinical and laboratory parameters, IL-6 had an adjusted odds ratio of 30.8 (95% CI = 1.1-728.3) (*P* = 0.046). A high CRP/albumin ratio of > 11.4 was associated with COVID-19 mortality [hazard ratio = 59.9 (95% CI = 7.4–488.3) (*P* < 0.0001)]. High CRP/albumin ratio had an adjusted hazard ratio of 26.5 (95% CI = 2.6-270.7) after adjustment of age and presence of co-morbidities (*P* = 0.006).

**Conclusion:**

IL-6 level could effectively discriminate COVID-19 severity. CRP/albumin ratio was an independent risk factor for 30-day mortality rate in patients with COVID-19. IL-6 and CRP/albumin ratio seem to be valuable biomarkers in evaluating the severity and prognosis of COVID-19, respectively.

## Background

Coronavirus disease 2019 (COVID-19) was announced in early December 2019. By genome sequencing, the virus was recognized [1]. From Wuhan City, the virus spread globally. The pandemic situation is declared by the World Health Organization [2]. In Egypt, the first case was detected on 14 February 2020, for a foreigner. After nearly 2 weeks, the Egyptian cases were detected [3].

COVID-19 clarified as a highly transmissible disease. The clinical features varied with disease severity. Most COVID-19 patients have non-severe manifestations and show a good prognosis. However, patients with severe disease may progress to pulmonary dysfunction, multiple organ dysfunction, and death [4]. COVID-19 related to a considerable mortality rate in older patients and cases had other morbidities [5].

In other coronaviruses, studies suggested that the inflammatory storm is a common finding [6]. Similarly, increases in the inflammatory markers like interleukin-6 (IL-6) and C-reactive protein (CRP) were described in COVID-19 [7]. Albumin levels decreased in the inflammatory conditions; reduced levels were confirmed in severe COVID-19 patients [8]. Hypoalbuminemia and high CRP/albumin ratio were previously linked to the mortality of various clinical conditions as critically ill patients [9].

To avoid the unnecessary or inappropriate utilization of the healthcare resources, early prediction of the severity of COVID-19 will be helpful. Severity prediction will also improve the prognosis by reducing the mortality rate [10]. Thus, this study aimed to evaluate the role of inflammatory markers in estimating the severity and predicting the prognosis of COVID-19. This study hypothesized that elevated values of IL-6, CRP, and CRP/albumin ratio at the time of COVID-19 diagnosis are associated with COVID-19 severity and mortality.

## Methods

### Study design

In June 2020, a cohort study was carried out on patients from Zagazig University Hospitals. The subjects’ participation was confirmed by signing the consent form either by the patient or the first degree relatives. The protocol of this study was affirmed by the Institutional Review Board of the Faculty of Human Medicine, Zagazig University (approval number: 6250). The patients were evaluated by full history and clinical examinations. The disease severity was assessed. At the time of diagnosis, measured and calculated laboratory tests were done. The primary outcome was 30-day mortality.

### Subjects

**Fig. 1:**
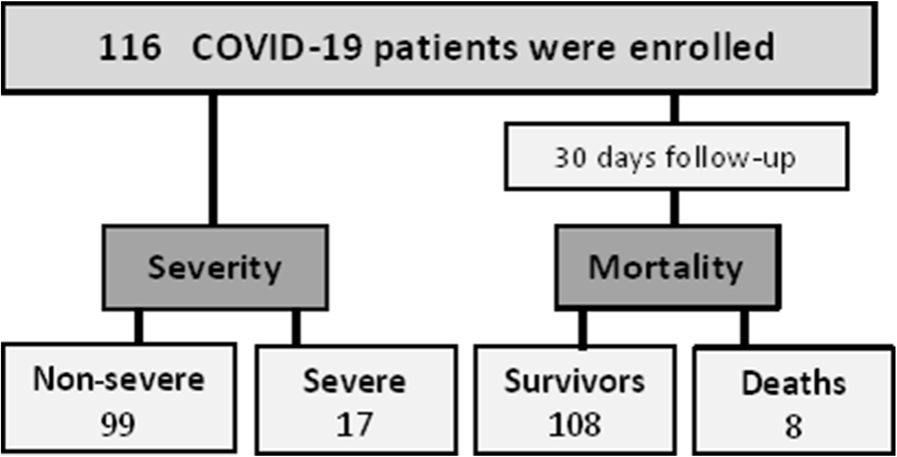
Study flowchart

The Epi Info program 6 (Atlanta, Ga, USA) was the utilized program for sample size estimation. The percentage of exposure and the outcome of either exposed or non-exposed groups were calculated following the study of Liu et al. [11] with 95% statistical power and 95% confidence limit. A total of 116 consecutive confirmed patients were included. The diagnosis and severity classification criteria were performed as stated by the management protocol of COVID-19 released by the Egyptian Ministry of Health and Population (2020). All included patients were diagnosed as COVID-19 positive ones based on the presence of viral nucleic acid in the nasopharyngeal swab. By disease severity, patients were sub-grouped. The patient considered as severe one if at least a single criterion of these criteria was present [increased respiration rate/minute > 30 or reduced oxygen saturation at room air < 92% or decreased partial pressure of arterial oxygen to fraction of inspired oxygen ratio < 300 mmHg or a chest lesion > 50% or a rapid advancement of chest damage within 24-48 h]. The critically ill patients were considered if their respiration rate/minute is > 30 or have reduced oxygen saturation < 92% or have decreased partial pressure of arterial oxygen to fraction of inspired oxygen ratio < 200 mmHg; despite oxygen therapy. Both severe and critically ill cases were included in the severe group. The patients were divided into two groups, including 99 patients in the non-severe group and 17 cases in the severe one (Fig. 1).

### Samples

In BD® Plus Plastic Serum vacutainer, blood samples were obtained. The plain vacutainers were allowed to clot for 30 min after collection. After this period, samples were centrifuged at 1200×*g* for 10 min to separate serum. The analysis of CRP and albumin was performed immediately. The remaining serum was transferred in 1.5-mL sterile microcentrifuge tubes and stored at −80 °C until IL-6 analysis.

### Methods

CRP and albumin were quantified using the CRP and albumin LR kits, respectively (Gesan Production Kit, Campobello di Mazara, Trapani, Italy). These biochemical tests were performed according to the manufacturer’s steps on the Microlab 300 (ELITech Group, France). IL-6 was quantified by Human IL-6 ELISA Kit (Bioassay Technology Laboratory, Shanghai, China) [Catalog No.: E0090Hu]. The assay steps were done according to the manufacturer’s procedure. The readings were done using the BioTek ELx800 Absorbance Microplate Readers (Bio-Tek Instruments, USA). The IL-6 levels were expressed in pg/mL. The precision coefficients of this IL-6 kit were an intra-assay CV of less than 10% and an inter-assay CV of less than 12%.

### Calculations

The calculated ratio was estimated using the following equation: “CRP/Albumin ratio = CRP level/Albumin level.”

### Statistical analysis

Data were checked by the Shapiro–Wilk test, a non-parametrically distribution was found. Mann-Whitney *U* test and chi-square test were used for two groups’ comparison. The performance of the laboratory test was evaluated by the receiver operator characteristic (ROC) curve. The optimal cutoff point was established by the highest Youden’s index. The Logistic Regression Analysis was utilized to clarify the predictors by measuring the odds ratio (OR) and its 95% confidence interval (CI). The outcome evaluation was performed by Kaplan-Meier survival analysis, log-rank tests, and the Cox-regression analysis. The accepted statistically significant value was a *P* value of less than 0.05. SPSS 16.0 (SPSS Inc., Chicago, IL, USA) was utilized.

## Results

A total of 116 COVID-19 patients were included in this study. Patients were assigned to the severe (14.7%) and non-severe (85.3%) groups. The demographic and clinical characteristics of patients according to severity were presented in Table 1. The severe group had significantly higher values of patients’ age and smoking percent than the non-severe group. The most prevalent symptoms were fever, cough, fatigue, and bone ache. The severe patients had a higher prevalence of dyspnea and chest tightness than that of non-severe patients. Severe patients were more likely to have co-morbidities in comparison with non-severe patients. In this cohort, eight patients (6.9%) died in the hospital. No mortality was detected among those with the non-severe disease.

**Table 1:** Demographic, clinical, and laboratory characteristics of the patients

Parameters	Non-severe (no.: 99)	Severe (no.: 17)	***P***
**Age, years**	36 [21-77]	54.5 [20-88]	0.001*
**Sex, male**	51 (51.5)	12 (70.6)	0.15
**Smoking**	6 (6.1)	4 (23.5)	0.018*
**Symptoms**			
• Fever	85 (85.9)	16 (94.1)	0.35
• Fatigue	22 (22.2)	5 (29.4)	0.52
• Bone ache	17 (17.2)	4 (23.5)	0.53
• Anosmia	55 (55.6)	4 (23.5)	0.015*
• Nausea or vomiting	3 (3)	1 (5.9)	0.55
• Diarrhea	5 (5.1)	2 (11.8)	0.28
• Abdominal pain	9 (9.1)	3 (17.6)	0.29
• Sore throat	9 (9.1)	3 (17.6)	0.29
• Cough	72 (72.7)	12 (70.6)	0.86
• Dyspnea	4 (4)	10 (58.8)	< 0.0001*
• Chest tightness	5 (5.1)	7 (41.7)	< 0.0001*
**Co-morbidities**			
• Diabetes	8 (8.1)	5 (29.4)	0.01*
• Hypertension	12 (12.1)	6 (35.3)	0.015*
• Coronary heart disease	2 (2)	3 (17.6)	0.003*
• Chest diseases	5 (5.1)	4 (23.5)	0.009*
• Thyroid diseases	0 (0)	1 (5.9)	0.015*
**Vital signs**			
• Respiratory rate, breaths/min	22 [20-23]	23 [21-30]	< 0.0001*
• Oxygen saturation, %	95 [94-97]	87 [72-94]	< 0.0001*
**Outcome**			
• Mortality	0 (0)	8 (47.1)	< 0.0001*
**Laboratory parameters**			
• IL-6, pg/mL	14.5 [6.8-47.5]	55.9 [20.5-123.5]	< 0.0001*
• CRP, mg/L	20.5 [6-73]	46 [18.5-183]	< 0.0001*
• Albumin, g/dL	4 [3-5.3]	3.5 [2.8-5]	0.024*
• CRP/albumin ratio	5.1 [1.6-18.7]	14.1 [5.8-48.2]	< 0.0001*

Data are expressed as median [range] or number (%)

^*^Significant

The laboratory findings of the studied patients at the time of diagnosis are presented in Table 2; the patients were sub-grouped according to the COVID-19 severity. Severe patients had higher levels of IL-6, CRP, and CRP/albumin ratio. On the other hand, the levels of albumin were decreased in severe ones.

**Table 2:** The performance criteria of the laboratory markers in discriminating the severity of COVID-19

Parameters	Cutoff	Youden’s index	AUC (95% CI)	Sensitivity	Specificity	PPV	NPP	Accuracy
IL-6, pg/mL	> 32.3	0.77	0.955 (0.912-0.997)	82.4%	94.4%	73.7%	96.9%	93.1%
CRP/albumin ratio	> 8.9	0.73	0.922 (0.862-0.981)	82.4%	90.9%	60.9%	96.8%	89.7%
CRP, mg/L	> 33.9	0.65	0.889 (0.805-0.973)	76.5%	88.9%	54.2%	95.7%	87.1%
Albumin, g/dL	< 3.8	0.42	0.672 (0.537-0.806)	82.4%	60.6%	26.4%	95.2%	63.8%

*AUC* area under the ROC; *CI* confidence interval; *CRP* C-reactive protein; *IL-6* interleukin 6

**Fig. 2:**
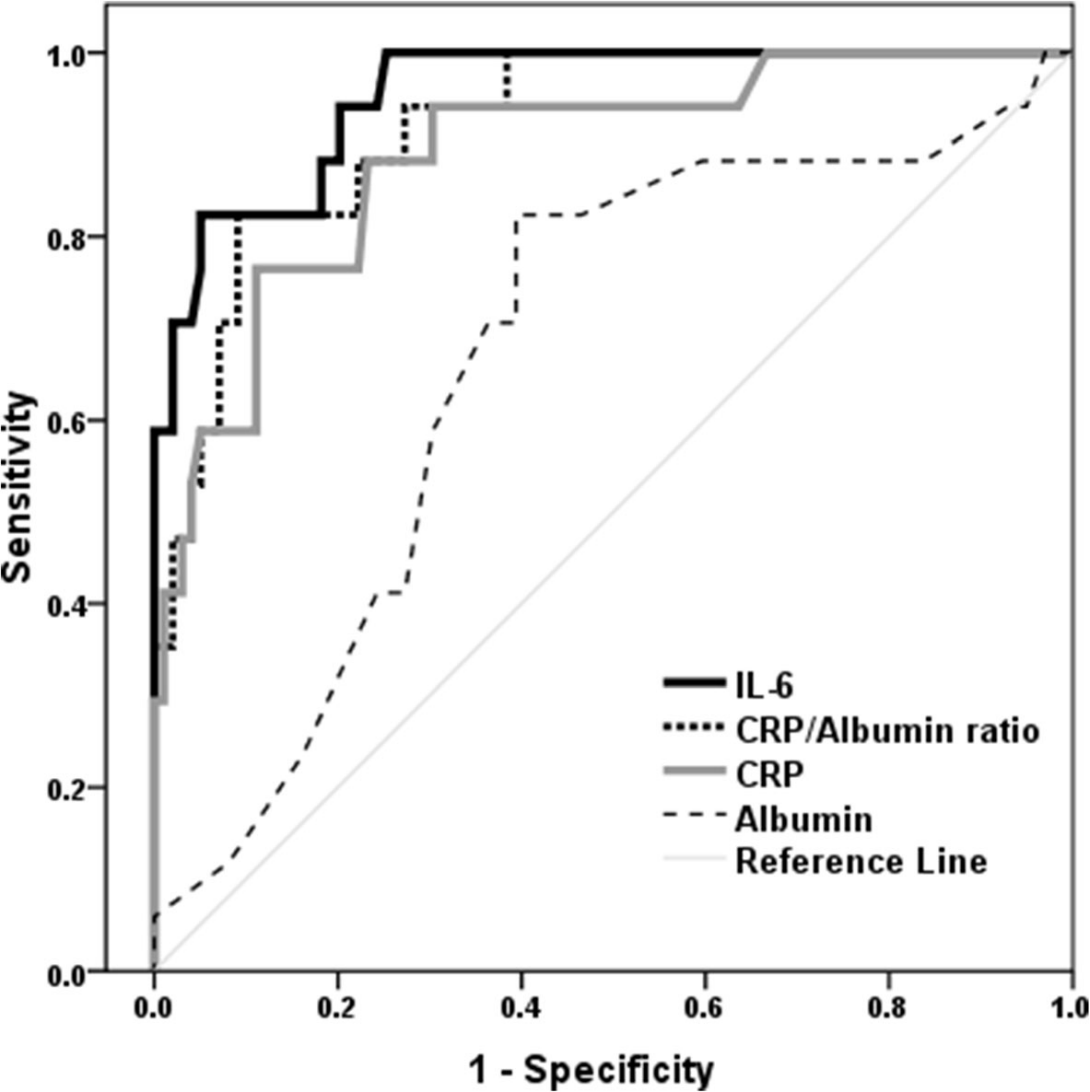
ROC curve of laboratory markers as predictors of COVID-19 severity

The role of laboratory tests in discriminating the severity of COVID-19 was evaluated. The ROC curve analysis was utilized. Non-severe patients were considered negative and severe patients as positive. The area under the ROC curve (ROC-AUC) was assessed (Fig. 2). IL-6 is the most sensitive predictor of COVID-19 severity. The performance criteria of the laboratory markers were presented in Table 2.

Further evaluation of the association between IL-6 and COVID-19 severity was performed by Logistic Regression Analysis (Table 3). IL-6 had OR of 87.7 (95% CI = 18.9-408.2) (*P <* 0.0001). Three models of adjustment were proposed. Model-1 adjustment utilizes the other significant laboratory tests includes CRP, CRP- albumin ratio, and albumin. In model 2, IL-6 had an adjusted OR of 79.1 (95% CI = 10.6-588.1). Model 2 was the adjustment for demographic and clinical data includes age, sex, smoking, hypertension, diabetes, chest diseases, thyroid diseases, cardiac diseases, dyspnea, chest tightness, and ansomia. IL-6 had an adjusted OR of 38.9 (95% CI = 2.6-589). Model-3 adjustment included all variables used in model 1 and model 2, and IL-6 had an adjusted OR of 30.8 (95% CI = 1.1-728.3) (*P* = 0.046). So, IL-6 can be an independent predictor of COVID-19 severity.

**Table 3:** IL-6 as a predictor of COVID-19 severity

IL-6	Odds ratio	95% Confidence interval	***P***
Crude	87.7	18.9-408.2	< 0.0001*
Adjusted			
• Model 1	79.1	10.6-588	< 0.0001*
• Model 2	38.9	2.6-589	0.008*
• Model 3	27.9	1.1-728.3	0.046*

Model 1, IL-6 adjusted to CRP, CRP/albumin ratio, and albumin

Model 2, IL-6 adjusted to age, sex, smoking, hypertension, diabetes, chest diseases, thyroid diseases, cardiac diseases, dyspnea, chest tightness, and anosmia

Model 3, IL-6 adjusted to all parameters included in model 1 and model 2

^*^Significant

During the follow-up period, eight patients died (6.9%). The follow-up period for the mortality data was varied between 1 and 30 days, with a median of 14 days. The overall survival of all patients was 93.1%. Regarding patient’s age, non-survivors had significant higher values than survivor (63.5 [31-88] and 37 [20-78] respectively; *P* = 0.003). Also, the prevalence of co-morbidities in non-survivors was significantly higher than survivors (87.5% and 31.5%, respectively; *P* = 0.001).

**Fig. 3:**
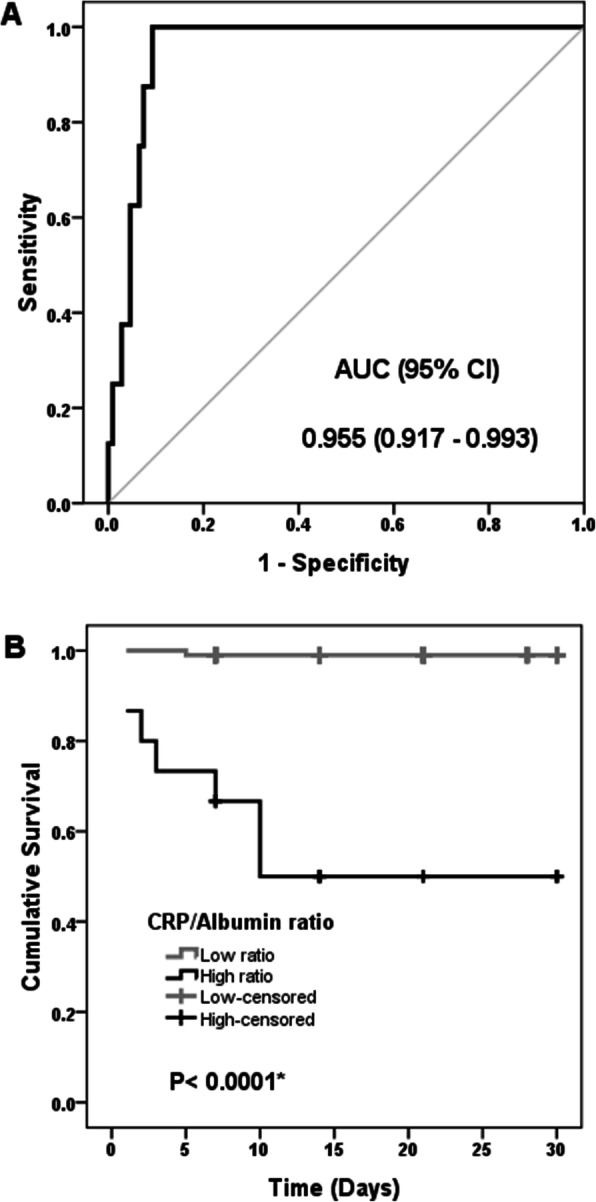
**a** ROC curve of CRP/albumin ratio as a predictor of COVID-19 mortality. **b** Kaplan-Meier survival curve according to the CRP/albumin ratio values

The importance of laboratory tests in detecting the COVID-19 mortality was assessed by ROC curve analysis. The laboratory tests had the highest ROC-AUC were CRP/albumin ratio, CRP, and IL-6. The ROC-AUC values were 0.955, 0.939, and 0.923, respectively. CRP/albumin ratio is the sensitive predictor of COVID-19 mortality (Fig. 3a). At CRP/albumin ratio value of 11.4 (the highest Youden’s index = 0.8), CRP/albumin ratio can predict mortality outcome. The COVID-19 patients were divided into two groups according to their serum CRP/albumin ratio values levels (low ratio group ≤ 11.4 and high ratio group > 11.4). The 30-day mortality rates were 0.99% and 46.7% in a group of low ratios and high ones, respectively. The overall survival was assessed by the Kaplan–Meier curve (Fig. 3b), it showed lower survival in patients with elevated CRP/albumin ratios (log-rank test, *P* <  0.0001). The Cox Regression Analysis showed that a high CRP/albumin ratio of > 11.4 was associated with COVID-19 mortality (hazard ratio = 59.9, 95% CI = 7.4–488.3, and *P* < 0.0001). High CRP/albumin ratio had an adjusted hazard ratio of 26.5 (95% CI = 2.6-270.7, *P* = 0.006) after adjustment of age and presence of co-morbidities. So, the CRP/albumin ratio could be a significant independent prognostic factor for COVID-19 mortality.

## Discussion

This study provided the baseline demographic, clinical, and laboratory characteristics of non-severe and severe COVID-19 patients. About 14.7% of the studied cohort fulfills the criteria of severe cases. The percentage of severe cases was comparable to the published risk of severity percentage [12]. The current study found that severe and non-survivor patients were elderly. The age of the patients is associated with worse disease progress and a higher mortality rate [13]. The smoking percentage was higher in severe cases. In the same line, Guo [14] revealed by a meta-analysis that smoking was associated with COVID-19 severity. Co-morbidities including hypertension, diabetes, cardiovascular, renal, hepatic, and chest diseases were prevalent in severe and non-survivor cases. This finding is in agreement with that of Yang et al. [15].

In this study, the most prevalent symptoms were fever, cough, fatigue, and bone ache. Dyspnea, chest tightness, and anosmia were the clinically significant discriminator between the non-severe and severe groups. The main symptom among COVID-19 cases was fever. Fever was presented in nearly eighty percent of the cases [16]. The second prevalent symptom was coughing [17]. The severe patient had a higher prevalence of dyspnea. Dyspnea seems to be a marker of severity [18]. Other detected symptoms include headache, fatigue, sore throat, and gastrointestinal symptoms were varying in prevalence [19].

The mortality rate in 30-day follow up was 6.9%. High mortality was significant in older age cases and the presence of co-morbidities. This mortality rate does not reflect the real population mortality rate due to the absence of population screening that underestimates the total number of patients [20].

This study aimed to evaluate the role of laboratory markers at the time of diagnosis in estimating the severity and predicting the prognosis of COVID-19. As far as the researcher knows, this study is the first to evaluate the role of CRP/albumin ratio in COVID-19 mortality prediction. This study showed that severe patients had significantly higher values of CRP, CRP/albumin ratio, and IL-6. On the other hand, the albumin levels decreased in the severe ones. ROC curve analysis revealed that the best discriminator of disease severity was IL-6 while CRP/albumin ratio was the best predictor of adverse outcome.

CRP is considered a sensitive biomarker of infection, inflammation, and tissue damage. During the acute inflammatory responses, the CRP level increases rapidly [21]. CRP suggested as an efficient marker in assessing COVID-19 severity [22]. This study revealed that CRP was associated with disease severity and clinical outcome. But IL-6 and CRP/albumin ratio performed better than CRP in predicting disease severity and adverse outcome.

The inflammatory reaction plays a prominent role in the pathophysiology of COVID-19. The pro-inflammatory cytokine increased in the peripheral blood of the patients [23]. The National Health Commission of China (trial 7th version) approves that peripheral blood IL-6 level increased during COVID-19 infection [24]. IL-6 was defined as an acute-phase inflammatory cytokine; its serum level reflects the degree of lung inflammation [25]. The current study assessed the IL-6 levels in COVID-19 patients. The patients with severe COVID-19 had higher levels of IL-6 in comparison with non-severe patients. Regards IL-6 diagnostic performance criteria, IL-6 seems to be a useful marker for early recognition of severe disease. IL-6 showed significantly predictive power even after adjustment to different models that include clinical and laboratory significant parameters. So, IL-6 can be considered as an independent predictor of COVID-19 severity. A meta-analysis was performed on nine studies to evaluate the role of IL-6 in assessing the COVID-19 severity. A significantly higher serum IL-6 levels were confirmed in patients with severe COVID-19 in comparison to non-severe ones [26]. These findings support that viral infection mediates lung injury via cytokines effects [27].

In this study, CRP levels alone can predict the mortality in patients with COVID-19. Significant hypoalbuminemia is detected in non-survivors COVID-19 patients. Reduced serum albumin level was associated with an increased mortality rate [28]. The CRP/albumin ratio was studied previously as a prognostic marker in critically ill patients with infections and malignancy [29]. This study showed lower survival in patients had high CRP/albumin ratios. However, albumin and CRP levels alternation may be due to the associated chronic illness [30]. Cox Regression Analysis showed that a high CRP/albumin ratio was associated with COVID-19 mortality even after adjustment to age and presence of co-morbidities. So, the CRP/albumin ratio could be a significant independent prognostic factor for COVID-19 mortality.

The current study had some limitations. First, this study was a single-center study, so further studies are required to include multiple centers with a much number of participants. Second, this study design was a cohort that lacks a control group. Lastly, the changes in the levels of the markers over time were not part of this study design, so further studies are recommended to assess their dynamic change. Future studies are suggested to confirm this study’s results.

## Conclusion

IL-6 level could effectively discriminate COVID-19 severity. CRP/albumin ratio was an independent risk factor for 30-day mortality rate in patients with COVID-19. IL-6 and CRP/albumin ratio seem to be valuable biomarkers in evaluating the severity and prognosis of COVID-19, respectively. High levels of the biomarkers require more attention that will enable better management.

## Data Availability

All the data of the current study are available from the corresponding author upon reasonable request.

## Abbreviations

CI: Confidence interval
COVID-19: Coronavirus disease 2019
CRP: C-reactive protein
IL: Interleukin
OR: Odds ratio
ROC: Receiver operating characteristic curve
ROC-AUC: Area under the ROC curve
